# Management of tumor volume changes during preoperative radiotherapy for extremity soft tissue sarcoma: a new strategy of adaptive radiotherapy

**DOI:** 10.2478/raon-2023-0056

**Published:** 2023-11-30

**Authors:** Marion Geneau De Lamarliere, Amélie Lusque, Justine Attal Khalifa, Vincent Esteyrie, Christine Chevreau, Thibaud Valentin, Dimitri Gangloff, Thomas Meresse, Louis Courtot, Philippe Rochaix, Bérénice Boulet, Eliane Graulieres, Anne Ducassou

**Affiliations:** Department of Radiation Oncology, Institut Claudius Regaud, Institut Universitaire du Cancer de Toulouse - Oncopole, Toulouse, France; Statistics department, Institut Claudius Regaud, Institut Universitaire du Cancer de Toulouse - Oncopole, Toulouse, France; Department of Radiation Oncology, Rodez, France; Department of Medical Oncology, Institut Claudius Regaud, Institut Universitaire du Cancer de Toulouse - Oncopole, Toulouse, France; Department of Surgery, Institut Claudius Regaud, Institut Universitaire du Cancer de Toulouse - Oncopole, Toulouse, France; Department of Surgery, Pierre Paul Riquet Hospital, Toulouse, France; Department of Pathology, Institut Claudius Regaud, Institut Universitaire du Cancer de Toulouse - Oncopole, Toulouse, France; Department of Imagery, Institut Claudius Regaud, Institut Universitaire du Cancer de Toulouse - Oncopole, Toulouse, France; Department of Engineering and Medical Physics, Institut Claudius Regaud, Institut Universitaire du Cancer de Toulouse – Oncopole. Toulouse, France

**Keywords:** soft tissue sarcoma, preoperative radiotherapy, adaptive radiotherapy, image guided radiotherapy, volumes changes

## Abstract

**Background:**

Using adaptive radiotherapy (ART), to determine objective clinical criteria that identify extremity soft tissue sarcoma (ESTS) patients requiring adaptation of their preoperative radiotherapy (RT) plan.

**Patients and methods:**

We included 17 patients with a lower extremity ESTS treated between 2019 and 2021 with preoperative RT, using helicoidal intensity-modulated RT (IMRT) tomotherapy, before surgical resection. We collected clinical, tumor parameters and treatment data. Repositioning was ascertained by daily Megavoltage computed tomography (MVCT) imaging. Using the PreciseART technology we retrospectively manually delineated at least one MVCT for each patient per week and recorded volume and dosimetric parameters. A greater than 5% change between target volume and planned target volume (PTV) dosimetric coverage from the initial planning CT scan to at least one MVCT was defined as clinically significant.

**Results:**

All 17 patients experienced significant tumor volume changes during treatment; 7 tumors grew (41%) and 10 shrank (59%). Three patients (18%), all undifferentiated pleomorphic sarcomas (UPS) with increased volume changes, experienced significant reductions in tumor dose coverage. Seven patients required a plan adaptation, as determined by practical criteria applied in our departmental practice. Among these patients, only one ultimately experienced a significant change in PTV coverage. Three patients had a PTV decrease of coverage. Among them, 2 did not receive plan adaptation according our criteria. None of the patients with decreased tumor volumes had reduced target volume coverage. Monitoring volume variations by estimating gross tumor volume (GTV) on MVCT, in addition to axial and sagittal linear tumor dimensions, appeared to be most effective for detecting reductions in PTV coverage throughout treatment.

**Conclusions:**

Variations in ESTS volume are evident during preoperative RT, but significant dosimetric variations are rare. Specific attention should be paid to grade 2–3 UPSs during the first 2 weeks of treatment. In the absence of dedicated software in routine clinical practice, monitoring of tumor volume changes by estimating GTV may represent a useful strategy for identifying patients whose treatment needs to be replanned.

## Introduction

Preoperative RT is one of the current standard of care for the management of extremity soft tissue sarcoma (ESTS), mainly in large tumors, requiring reconstructive surgery or possible R1 resection.^1,2^ This strategy allows to reduce the irradiation field, uses lower doses, reduces late toxicities^3^ and facilitates the delineation.^4,5^

Preoperative RT specifically confronts physicians with variations in tumor volume, enhanced particularly since the advent of image guided radiotherapy (IGRT), the development of volume repositioning systems such as cone-beam computed tomography scan (CBCT) and megavoltage computed tomography (MVCT). Approximately half of the patients treated with neoadjuvant radiotherapy, reported in the literature, present with a significant variation in tumor volume during the course of treatment.^6^ These variations in volume require the modification of planned treatment in approximately 8 to 30% of cases.^6,7,8,9,10,11^ Offline adaptive radiotherapy (ART) is currently the most appropriate approach to address gradual sarcoma anatomy changes, which may otherwise introduce interfractional errors.

Several authors have defined a tumor size variation of >1cm in any direction or recurrent incorrect and unacceptable repositioning, to trigger a plan adaptation.^10^ Others have opted to define tumor volume criteria.^11^ But there is currently no consensus on any objective cutoff thresholds which would prompt an adaptation of the previously planned treatment.

The aim of this retrospective study was to determine objective criteria to identify patients requiring plan adaptation in the clinical setting. We initially considered the threshold of a 5% decrease in planned target volume (PTV) coverage to be unacceptable for optimal treatment, with the rationale of ultimately finding an objective criterion that could be monitored throughout RT treatment.

## Patients and methods

This retrospective monocentric observational study was approved by and conducted in accordance with local ethic committee requirements (# F20210208164425). All procedures performed in studies involving human participants were in accordance with the 1964 Helsinki declaration and its later amendments or comparable ethical standards. We reviewed data from computer file records of patients treated between August 2019 and January 2021. Patient inclusion criteria were defined as: (i) a localized lower extremity ESTS, (ii) age of >18 years, (iii) preoperative radiotherapy or concomitant radio-chemotherapy (RTCT) with helicoidal intensity-modulated radiotherapy (IMRT) on tomotherapy. Patient treatments were planned on the Accuray® precision treatment planning system.

All dosimetric plans complied with our institutional optimal coverage criteria: 95% of PTV had to be covered by 95% of the prescribed dose, whilst respecting healthy organ dose limits.

Daily high-energy 3D IGRT (MVCT) image guidance was performed for all patients. Data was retrospectively uploaded into the tomography integrated PreciseART® adaptive radiation therapy software. The same radiation oncologist manually contoured each individual patient's gross tumor volume (GTV), clinical target volume (CTV) and PTV on at least one MVCT per week.

According to our departmental practice, the technicians reported any significant change in tumor volume (specifically a linear variation of >1cm in any direction), weight loss or any other incongruous parameter, compared to the initial planning CT (CTs1), to the radiation oncologist who then decided whether or not to adapt the plan. If a new plan was initiated, this involved performing a new CT simulation scan (CTs2), determining the GTV/CTV/PTVs, delineating the critical structures and recalculating the dosimetry from scratch. This new tailored plan was then applied to the patient in the next few days, and patients continued the treatment with the old plan waiting the new one.

The co-recording and fusion of the CT simulation scan (CTs1) with the contoured MVCT allowed us to determine axial and sagittal linear tumor dimensions, tumor volume, and dose coverage of the GTV and PTV over time. Since tumor volume in clinical practice cannot be obtained without re-delineation, we estimated it from axial and sagittal linear tumor dimensions using the formula ת × r2 × h (where r: axial diameter/2 and h: sagittal height), which assumes that the tumor is a cylinder.

A change in target volume of 5% at any time during treatment was defined as significant. We defined any change in dosimetric coverage of the PTV of 5% between the initial planning CT and the last MVCT as unacceptable.

### Statistical analysis

Continuous variables are represented as medians or means with a range (minimum–maximum), and categorical variables as frequencies and percentages.

For each patient, the percentage change of individual parameters was plotted over the six weeks of radiotherapy and the plan adaptation (if applicable). Percentage changes were calculated from the initial planning CT (CTs1) at each MCVT. If a plan adaptation was performed, the percentage change at each MVCT post adaptation was calculated from the new CT simulation scan (CTs2) for dosimetric data. The percentage change from CT1 at each MVCT in GTV was plotted over the six weeks of radiotherapy according to tumor histology in patients with increased or decreased GTV. Statistical analysis was carried out using Stata version 16 (StataCorp LLC, College Station, TX).

## Results

Patients, tumor, and treatment characteristics are presented in Table 1.

**TABLE 1.** Patients, tumor and treatment characteristics

**Characteristics:**	**n (%)**
**Sex**	
Male	12 (71)
Female	5 (29.4)
**Age at initial diagnosis, years**	
Median (range)	69 (43–90)
**Dimension, cm (diagnostic MRI)**	
Median (range)	12.8 (6–30)
**Pathology**	
Undifferentiated pleomorphic sarcoma (UPS)	8 (47)
Myxoid liposarcoma (MLS)	6 (35)
Dedifferentiated liposarcoma (DLS)	2 (12)
Pleomorphic rhabdomyosarcoma (PRS)	1 (6)
**Grade**	
1	6 (35)
2	7 (41)
3	4 (24)
**RT schedule (total dose, dose fraction)**	
50.4 Gy, 1.8Gy	5 (29)
50 Gy, 2Gy	10 (59)
45 Gy, 3 Gy	1 (6)
70 Gy, 2 Gy*	1 (6)
**GTV on CTs1 (ml)**	
Median (range)	381 (84–2908)
**PTV on CTs1 (ml)**	
Median (range)	1373 (587–5793)
**D95%PTV on CTs1 (%)**	
Median (range)	97.1 (96–99.1)
**Interval between CTs1 and MVCT 1 (days)**	
Median (range)	13 (9–17)
Neoadjuvant CT**	
No CT	10 (59)
Adriamycin ifosfamide ((doxorubicin 20 mg/m^2^ and ifosfamide 2500 mg/m^2^ day 1, 2 and 3 for 4 cycles (21-day cycle))	6 (35)
Adriamycin (doxorubicin 75 mg/m^2^ day 1 for 4 cycles (21-day cycle))	1 (6)

CTs1 = CT simulation scan; D95%PTV = dose received by 95% of the PTV volume; MRI = Magnetic Resonance Imaging; MVCT1 = first fraction of RT

GTV = gross tumor volume; MVCT = megavoltage computed tomography;PTV = planned target volume; RT = radiotherapy

* Patient initially scheduled to have preoperative 50 Gy in 25 fractions but deemed inoperable, leading to a modification of the prescription.

** 6 patients received 3 cycles, 1 patient had 4 cycles

All patients had a lower limb sarcoma. There were 8 undifferentiated pleomorphic sarcomas (UPS), 6 myxoid liposarcomas (MLS), 2 dedifferentiated liposarcomas (DLS) and 1 pleomorphic rhabdomyosarcoma (PRS). Majority of patients received 50 to 50.4 Gy delivered in 25 to 28 fractions.

The median interval between the end of RT and surgery was 57 days (range 32–131 days), all resections except one were R0 (adequate margins). Seven patients among the 14 patients for whom post-surgical data was available developed an acute surgical complication: 3 scar disunions, 2 infections, 1 deep vein thrombosis and 1 lymphocele. The 3 scar disunions all occurred in patients with shrinking tumors, and despite a skin flap reconstruction in 2 of the patients, during the sarcoma surgery.

Volumetric and dosimetric changes are presented in Table 2

**TABLE 2.** Gross tumor volume (GTV) and planned target volume (PTV) volumes variations, the largest GTV and D95% PTV changes during the course of treatment

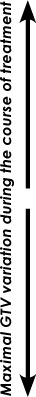	**Pathology**	**Vol GTV CTs1 (ml)**	**Vol PTV CTs1 (ml)**	**Vol GTV last MVCT**	**Vol PTV last MVCT**	**Largest GTV vol change % (week)**	**Largest D95%PTV change % (week)**
	UPS	674	1373	560	1150	**+36.4 (2)**	−12 (2)*
	PRS	239	1037	238	966	**+33.7 (2)**	−3.1 (1)
	UPS	381	1432	486	1706	**+32.3 (5)**	−20.1 (6)*
	UPS	2546	5292	3243	6263	**+27.4 (6)**	+ 1 (4)
	DLS	2908	5793	3444	6628	**+18.9 (4)**	−4.6 (3)
	UPS	1356	3534	1319	3446	**+11 (1)**	−2.1 (1)
	UPS	234	999	246	1086	**+7.9 (2)**	−14.1 (4)*

	UPS	140	587	144	598	**−7.2 (5)**	−3.4 (2)
	UPS	826	2073	781	2031	**−9.2 (1)**	−7.6 (4)
	DLS	531	1679	461	1509	**−13.2 (6)**	−1.3 (3)
	MLS	258	952	184	805	**−28.9 (6)**	−1.3 (2)
	MLS	415	1514	233	1098	**−43.8 (6)**	+ 1.6 (6)
	MLS	192	816	104	630	**−46.2 (6)**	−2 (6)
	MLS	228	629	122	398	**−46.4 (6)**	+0.4 (4)
	MLS	1062	3084	568	2158	**−46.5 (6)**	+ 1.14 (5)
	UPS	208	961	96	602	**−52 (6)**	−0.9 (1)
	MLS	84	606	44	548	**−52 (5)**	−7 (3)

DLS = dedifferentiated liposarcoma; MLS = myxoid liposarcoma; PRS = pleomorphic rhabdomyosarcoma; UPS = undifferentiated pleomorphic sarcoma

Re-planned patients are shown in gray, patients with PTV under-coverage are marked with a star (*)

All patients experienced a GTV volume variation of >+/−5% during treatment, a decrease in GTV in 10 patients (59%) and an increase in 7 patients (41%). Among these latter 7 patients, GTV initially increased in 3 patients before significantly decreasing. Mean maximal tumor volume changes were +24% (range +8% to +36%) for the GTV increases and −35% (range −7% to −52%) for GTV decreases. GTVs predominantly increased during the 1st week (78%) and decreased during the 3rd week (55%) of treatment.

Significant PTV volume changes, although less substantial (+16% (range +5% to +29%) for increases and −20% (range −7% to −37%) for decreases), followed the same trends as GTV changes, except for one patient whose PTV was very close to the skin, which presumably prevented its expansion.

**FIGURE 1.**
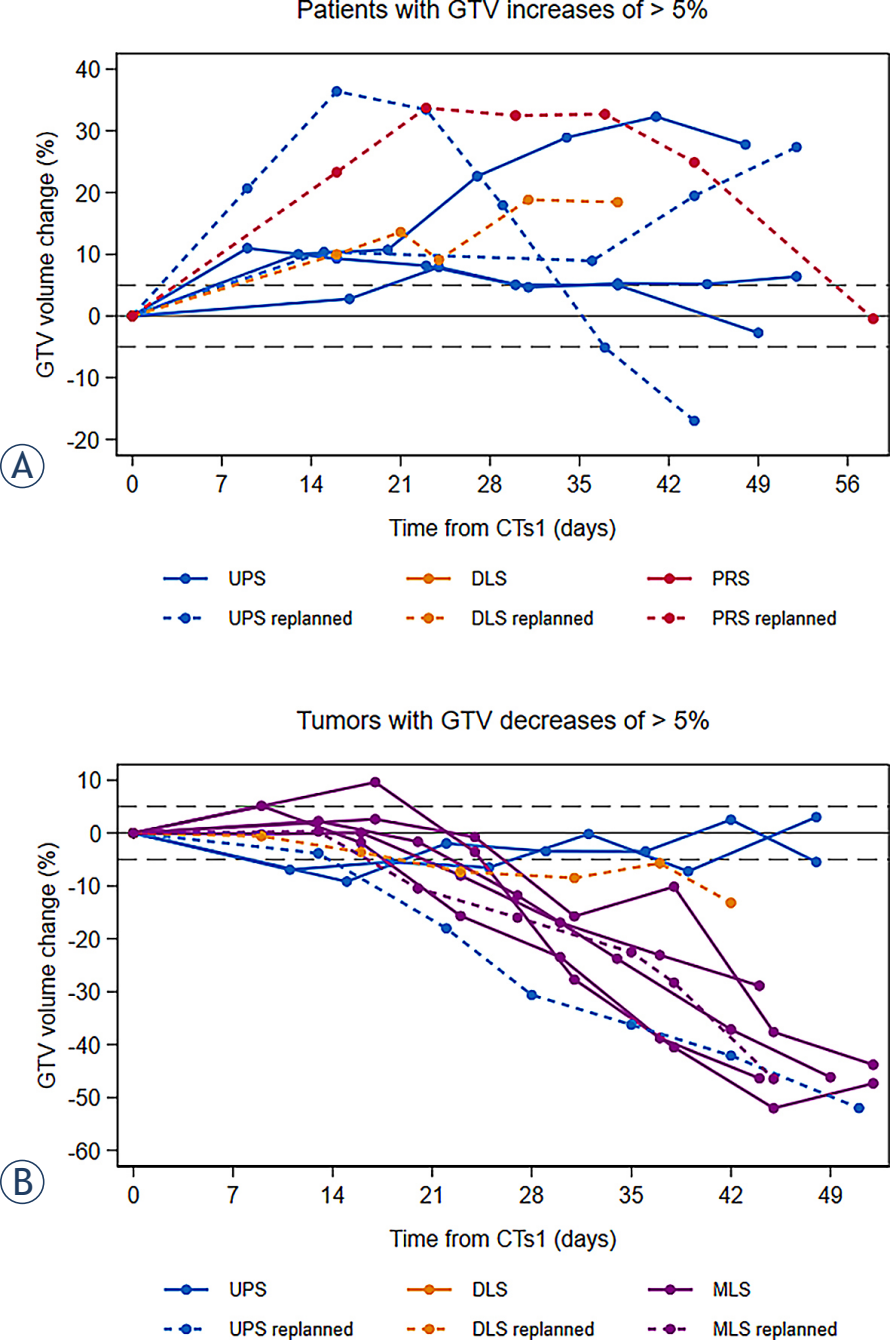
Percentage of maximal GTV changes during treatment: patients with significant gross tumor volume (GTV) increase **(A)**, patients with significant GTV decrease (B)*. Day 0 corresponds to CTs1. Each color represents a different histology subtype; replanned patients appear as dotted line. DLS = dedifferentiated liposarcomas; MLS = myxoid liposarcomas; PRS = pleomorphic rhabdomyosarcoma; UPS = undifferentiated pleomorphic sarcomas * Only the largest variation is shown for patients who presented with both an increase and a decrease in tumor size during the course of treatment.

Axial and sagittal GTV dimensions varied less significantly and remained stable in 24% and 29% of patients respectively. Variations based on the calculated estimate of the GTV (GTVr = ת × r2 × h) were overall well correlated with those of the actual GTV: 7 tumors increased (47%) and 9 tumors decreased (53%), 1 remained stable, at the time of maximal GTV changes. Volume variations and the most substantial GTV changes are shown in Table 2. Figure 1 shows GTV volume changes between CTs1 and the end of treatment in patients who presented a significant GTV increase (a), and in those who presented a significant GTV decrease (b), according to tumor histology subtypes.

MLSs decreased in size during RT, whereas 63% of UPS increased. Similarly, all grade 1 tumors decreased whilst 63% of grade 2 and 3 sarcomas increased.

We evaluated whether GTV variations correlated with changes in axial and sagittal linear tumor dimensions (Table 3).

**TABLE 3.** Correlations between GTV volume variations and dosimetric and clinical characteristics

		**Maximal GTV change**	

		**Decrease (n=10)**	**Increase (n=7)**
GTV largest axial axis*	Decrease	7 (70%)	0
	Stable	3 (30%)	3 (43%)
	Increase	0	4 (57%)
GTV largest sagittal axis*	Decrease	8 (80%)	0
	Stable	2 (20%)	3 (43%)
	Increase	0	4 (57%)
Estimated GTV volume	Decrease	9 (90%)	0
	Stable	1 (10%)	0
	Increase	0	7 (100%)
PTV volume	Decrease	8 (80%)	0
	Stable	2 (20%)	0
	Increase	0	7 (100%)
D95% PTV	Decrease	0	3 (43%)
	Stable	10 (100%)	4 (57%)

Surgical complications**	Yes	3 (33%)	4 (80%)
	No	6 (67%)	1 (20%)
Histology subtypes	UPS	3 (30%)	5 (71%)
	DLS	1 (10%)	1 (14%)
	MLS	6 (60%)	0
	PRS	0	1 (14%)
Histology grade	1	6 (60%)	0
	2	2 (20%)	5 (71%)
	3	2 (20%)	2 (29%)
Neoadjuvant CT	Yes	3 (30%)	4 (57%)
	No	7(70%)	3 (43%)

* Axial and sagittal linear tumor dimensions were recovered from the same slice for each MVCT, the slice that contained the largest tumor axial and sagittal axis on the CTs1.

** Data available for 14 patients: one patient had not been operated; the two others had not been operated when the analysis was performed.

GTV = gross tumor volume; MVCT = megavoltage computed tomography; PTV = planned target volume

The axial and sagittal tumor dimensions were stable when the GTV variation was at its maximum but varied significantly during subsequent MVCTs. A lag between significant GTV variation and significant changes in axial or sagittal linear tumor dimension was observed in 59% of cases. The estimated GTV also correlated well with the GTV over time.

The dose received by 95% of the PTV (D95%PTV) remained satisfactory for most patients. The largest D95%PTV changes are presented in Table 2. Three patients (18%) exhibited a significant drop in PTV coverage (mean maximal D95%PTV change of −15.3% (range −14.1% to −20.1%)). All occurred within the first 2 weeks of treatment at a mean of 4 fractions, following an average increase in GTV and PTV volumes of +17% and +11% respectively. All 3 patients had UPS, only one had a plan adaptation, because of an increase in axial length (+1.3 cm). The plan adaptation was performed in week 4 and restored an appropriate dosimetric plan. The remaining two patients sustained PTV under-dosage until the end of treatment. Volumetric and dosimetric data for these 3 patients are presented in Table 4. All 3 patients experienced significant increases in GTV, estimated GTV and PTV volumes. Conversely, sagittal and axial linear tumor dimensions were less informative. In patient 16, GTV and PTV did not immediately increase when the reduction in PTV coverage became apparent (Table 4).

**TABLE 4.** Dosimetric and clinical data of patients with significant reductions in PTV coverage

**Dosimetric data at first MVCT showing a variation of volume**					**Concomitant volume changes**			Clinical dimension change*	**D95% PTV on last MVCT**

**D95% PTV on CTs1**	**Fraction n°**	**Variation of D95 coverage (%)**	**New D95% PTV**	**D95% GTV**	**GTV (%)**	**PTV (%)**	**Estimated GTV(%)**		
99%*	6*	−11.8*	87.22%*	72.96%*	+36.4*	+22.8*	+27.6*	+ 1.3 cm (ax)*	98.71%*
96.1%	6	−10.4	**86.49%**	98.03%	+10.7	+6.2	+12.2	+ 1 cm (sag)	**77.07%**
98.2%	1	−5.9	**92.39%**	98.91%	+2.8	+3.3	+2	No	**94.29%**

Significant reductions in coverage are indicated in bold. The patient who had a plan adaptation is marked with a star (*).

* The “clinical dimension change” corresponds to the largest visible variation, all slices combined.

GTV = gross tumor volume; MVCT = megavoltage computed tomography; PTV = planned target volume

No quantitative variation in volume or dimension could precisely identify which of the 7 patients with growing tumors were going to have insufficient PTV coverage.

Among the 10 patients with shrinking tumors, the bone near-maximum absorbed dose (D2%) increased by an average of 1.6% (range 0.4 to 2.9%) in 9 patients. The D2% delivered to the joint increased by an average of 17% (range 1 to 65%) in 7 patients.

### Adaptive radiotherapy

Seven patients had a plan adaptation, based on empirical clinical criteria defined by the department: 4 tumor size increases, 2 tumor shrinkages1 loss of weight. Dosimetric and clinical data of patients with a plan adaptation are presented in Table 5.

**TABLE 5.** Dosimetric and clinical data of patients with a plan adaptation

**Dosimetric data just before plan adaptation**					**Evolution of D95%PTV**		

Clinical Reason*	**Fr n°**	**% GTV vol change**	**%PTV vol change**	**OAR constraints**	**At CTs1**	**Before re-planning**	**At CTs2**
Ax. increase (+ 1.3 cm)	13	+ 33	+ 22	Better	99.1%	**88%**	97.5%
Ax. increase (+ 0.9 cm)	4*	+ 9	+ 9	Better	98.1%	93.5%	96%
Sag. increase (+ 1.2 cm)	1	+ 23	+ 10	Better	96.8%	93.7%	95.9%
Sag. increase (+ 1 cm)	11	+ 17	+ 12	NA	96.9%	97.6%	97.6%
Ax. decrease (− 1.8 cm)	13	− 36	− 21	Bone Dm + 1.8%	96.9%	97.2%	98.7%
Sag. decrease (− 2 cm)	17	− 28	− 17	Bone Dm + 1%	96.4%	97.5%	96.5%
Weight loss	21**	− 13	− 10	Bone Dm + 30.7%	96.4%	97.1%	96.9%

Ax = axial; Bone Dm = average dose to the bone; CTs1 = CT computed tomography simulation scan; CTs2 = new Fr n° = fraction number; GTV = gross tumor volume; NA = result not available; OAR = organ at risk; PTV = planned target volume; Sag = sagittal

* The “clinical reason” data of axial and sagittal linear tumor dimensions of patients who had a plan adaptation corresponds to the largest visible variation, all slices combined.

The decision to adapt a plan was predominantly made the 3rd week, at a mean dose of 21.6 Gy (range 0 Gy to 42 Gy). Retrospectively, PTV coverage remained adequate for 6 out of the 7 patients. In the two patients with tumor shrinkage, PTV coverage was stable and OAR constraints were respected. On the CTs2, an average increase of 17.2% (range −41 to 42%) in tumor volume was observed. The new plans resulted in a 4.7% gain of PTV coverage, all OAR constraints were improved by making a new treatment planning.

### Clinical considerations

Six MLS were included in our study. All tumors shrank during treatment, by 44% on average. Their dose coverage remained relatively stable with a −1.2% mean maximal D95% PTV change during the treatment. Only one of the cases presented a significant decrease in PTV coverage (−7% at week 3). Graphs for each of the 17 patients showing the evolution of tumor dimensions and volumes throughout treatment are included in the appendix.

## Discussion

The current trend in the treatment of patients with locally advanced ESTS is the use of neoadjuvant RT. This practice forces physicians to consider the management of tumor volume changes during radiotherapy. The 17 patients included in our study and treated by helical IMRT on tomotherapy, all presented a significant volume change at some point during their treatments. We observed more tumor size reductions (59%) than increases (41%).

ESTS volume changes during preoperative radiotherapy are obvious and widely described in the literature. However, the magnitude and frequency of changes vary substantially due to variations in the criteria adopted, the histology subtype and the nature of the neoadjuvant treatment administered. Abu-Hijlih *et al*. reported 61% of tumor volume variations, the majority decreases 57%. The comparison was only based on the last CBCT and therefore excluded any short-term fluctuations.^9^ Under similar conditions, 18% of our patients would have been considered stable (with 59% reductions but only 23% increases). Conversely, Dickie *et al*. who only considered patients with a plan adaptation, noted more tumor parameter increases than decreases (64% vs 36%).^10^ Betgen *et al.* only found 52% volume variations between the start and the end of treatment although they applied the same variation criteria as our study. Surprisingly, 60% of their tumors were MLS, which are known to significantly decrease in size during treatment. Betgen *et al*. observed a 33% tumor shrinkage among the MLSs. Furthermore, their 5 tumors that increased in size, exhibited a mean GTV change that was lower than ours (+14% vs +22%).^11^ Histology subtype was a risk factor for tumor volume changes. In our study, UPSs tended to increase in size, whilst MLSs consistently decreased. The high frequency of myxoid liposarcomas (6 patients, 35%) in our cohort, which are known to decrease in size after treatment, may explain the higher frequency of tumor shrinkages that we observed. Authors such as Dickie *and al*., who considered that the cohort of tumors that increased in size predominantly consisted of UPS, have previously reported the increase in UPS size during treatment. Pitson and Magierowski *et al.* also compared MLS to UPS behavior.^12,13^ They analyzed volume changes of 16 MLS and 16 UPS. The mean pretreatment and post treatment volume of the MLS was 415 and 199 cm^3^, respectively (P = <0.0001). The mean pretreatment and post treatment volume of the UPS was 264 and 273 cm^3^, respectively (p = 0.804). These studies confirmed that MLS decrease during RT, meanwhile UPS are stable or grow.

Concurrent neoadjuvant chemotherapy also increased tumor volume changes. To the best of our knowledge, our study is the first to include patients treated with concurrent radio-chemotherapy RTCT. However, we did not observe a significant correlation between GTV changes and the administration of neoadjuvant CT.

Most of our adaptive interventions occurred during the 3rd week of treatment, at a mean of 11 fractions, which is consistent with Abu-Hijlih and Dickie *et al*. In 2013, Betgen *et al*. proposed an optimal time point for adaptive intervention: after week 1 for non-MLS patients and after week 3 for MLS patients.^11^ This is totally consistent with both the clinical and dosimetric variations that we observed over the whole treatment period, since we noted that significant volume changes occurred predominantly during the 3rd week for tumors that were shrinking whilst significant tumor volume increases appeared earlier, during the 1st week. Grade 2 or 3 UPS behavior seemed to be more difficult to anticipate, requiring specific attention during the first week of treatment.

Despite all of these tumor volume variations, a reduction in PTV coverage was ultimately not a very frequent occurrence. Over the whole treatment period, only 3 patients presented with a decrease in D95%PTV >5%, all were growing UPS tumors and these changes occurred during the first 2 weeks of treatment.

In shrinking tumors, we did not observe any changes in PTV coverage >5%, or significant overdosing of OARs. Our results are consistent with those of Dickie *et al*. Among their 8 patients who had a 2nd CT scan because of tumor shrinkage, no significant change in the mean dose to the PTV and no significant increase in the dose to the adjacent bone were observed.

In the 7 patients (41%) who were replanned according to our departmental protocol, only one patient presented with a significant decrease of D95%PTV. Our plan adaptation rate is higher than that reported in the literature: Abu-Hijlih *et al*. adapted the plan of 17% of their 23 patients, O’Sullivan *et al*. 15% of patients in the phase II IG-IMRT trial and Rick L. Hass *et al*. only 8%.^6,14^ There are several reasons for this. Firstly, some authors only re-planned tumors that were growing whereas we also included the 3 tumors that shrank. Secondly, as already highlighted, we observed more volume variations and overall, more pronounced amplitudes in our cohort compared to previous studies, perhaps due to the chemotherapy. Our institutional practice also required patients to be replanned based on clinical data, often when axial or sagittal linear tumor dimensions changed by >1cm. In these cases, patients directly benefited from a new planning CT with complete treatment plan adaptation, potentially resulting in more replanifications than in other centers where plan adaptation was performed only in cases of effective PTV under-coverage.^10^

As plan adaptation is time-consuming, we tried to evaluate better clinical tools to identify patients that would benefit from plan adaptation. Since variations in axial and sagittal linear tumor dimensions occurred later compared to variations in GTV, PTV and estimated GTV volumes, we found estimated GTV volume (which considers the tumor as a cylinder and is estimated based on only 2 dimensions; axial diameter and sagittal height) to be a sensitive clinical parameter, that is easy to calculate, allowing weekly volume changes to be evaluated on MVCT or CBCT.

Results from our small series of patients may be useful to modify ART practices. We observed that tumors at high risk of volume increases (specifically UPSs) might be expected to have a more substantial reduction in PTV coverage than others. For this type of tumor histology, we therefore suggest a detailed follow-up during the first 2 weeks of treatment, measuring axial and sagittal linear tumor dimensions, calculating an estimated GTV on repositioned imaging, and comparing this with the initial GTV estimate.

Plan adaptation does not seem useful in shrinking tumors, due to the apparent absence of consequences on PTV or OAR coverage. However, the occurrence of 2 scar disunions in our cohort calls for caution. The lack of specific data on healthy soft tissue or skin doses prevented us from pursuing this analysis.

Our study had several limitations, mainly its small series of patients. Then, limitations come from the ART procedure itself. Firstly, dedicated ART software cannot be easily accessed in the practice, due in part to its substantial cost. With the recent development of this technique, many more software options to address this shortfall are now emerging. Some of these use CBCT with appropriate contrasts for the delineation, most include elastic registration and automatic contour delineation. An innovative alternative, MRlinac®, uses 4D-MR imaging with minimal latency times allowing better visualization of soft tissue and on-line ART. Deep learning-based dose prediction, as recommended for offline plan adaptation, is considered an appropriate solution for real-time dose reconstructions.^15,16^ We used the PreciseART software (Accuray®), which allows ART to be performed on MVCT. Although the software has elastic registration and automatic delineation capabilities, these techniques have not yet been validated and we opted for rigid registration and predominantly manual delineation. Under these conditions, the practice of ART is time-consuming and rigid registration is known to be suboptimal for dose reconstruction studies. These limitations were recently described in the POP-ART study in which 177 centers from 40 countries responded to a questionnaire about their ART practices. ART was used by 61% of respondents; the plan adaptation decision was made “ad-hoc” (without protocol) in the vast majority of cases (69%) and was predominantly performed offline. Only 10% used MR imaging, which allowed daily online plan adaptation. Nineteen percent of respondents used their in-house software because commercially available software lacked functionality. In addition, only 4 centers in this study specifically adapted treatment to sarcomas, all of them, like us, by ad hoc off-line plan adaptation.^17^

## Conclusions

Variations in tumor volume are apparent during preoperative ESTS-RT, but their dosimetric consequences are rare and mostly affect patients with tumor volume increases. To identify patients at risk of significant variations in PTV coverage, special attention should be paid to grade 2 and 3 UPS patients during the first 2 weeks of treatment. Monitoring volume changes by calculating an estimated GTV volume in addition to monitoring axial and sagittal linear tumor dimensions throughout radiation therapy may prove to be a good approach for detecting any significant reductions in PTV coverage.

## Supplementary Material

Supplementary Material Details

## References

[1] Beane JD, Yang JC, White D, Steinberg SM, Rosenberg SA, Rudloff U (2014). Efficacy of adjuvant radiation therapy in the treatment of STS of the extremity: 20-year follow-up of a randomized prospective trial. Ann Surg Oncol.

[2] O’Sullivan B, Davis AM, Turcotte R, Bell R, Catton C, Chabot P (2002). Preoperative versus postoperative radiotherapy in STS of the limbs: a randomised trial. Lancet.

[3] Davis AM, O’Sullivan B, Turcotte R, Bell R, Catton C, Chabot P (2005). Late radiation morbidity following randomization to preoperative versus postoperative radiotherapy in extremity soft tissue sarcoma. Radiother Oncol.

[4] Le Péchoux C, Moureau-Zabotto L, Llacer C, Ducassou A, Sargos P, Sunyach MP (2016). RECORAD 2016: Radiothérapie des sarcomes des tissus mous de l’adulte. Cancer Radiother.

[5] Wang D, Bosch W, Roberge D, Finkelstein SE, Petersen I, Haddock M (2011). RTOG Sarcoma Working Group Consensus on the GTV and CTV for preoperative RT. Int J Radiation Oncology Biol Phys.

[6] Haas RL, van Beek S, Betgen A, Ali S, Schneider CJ, Diddens FH (2019). Substantial volume changes and plan adaptations during preoperative radiotherapy in extremity soft tissue sarcoma patients. Practical Radiat Oncol.

[7] Mc Nair H, Buijs M (2019). Image guided radiotherapy moving towards real time adaptive radiotherapy; global positioning system for radiotherapy?. Tech Innov Patient Support Radiat Oncol.

[8] Stankiewicz M, Li W, Rosewall T, Tadic T, Dickie C, Velec M (2019). Patterns of practice of adaptive re-planning for anatomic variances during cone-beam CT guided radiotherapy. Tech Innov Patient Support Radiat Oncol.

[9] Abu-Hijlih R, Mheid S, Abuhijla F, Asha W, Mohamad I, Alrashdan A (2019). Adaptive radiotherapy in patients receiving neoadjuvant radiation for soft tissue sarcoma. Rep Pract Oncol Radiother.

[10] Dickie C, Parent A, Griffin AM, Wunder J, Ferguson P, Chung PW (2017). The value of adaptive preoperative radiotherapy in management of soft tissue sarcoma. Radiother Oncol.

[11] Betgen A, Haas RLM, Sonke JJ (2013). Volume changes in soft tissue sarcomas during preoperative radiotherapy of extremities evaluated using cone-beam CT. J Radiat Oncol.

[12] Pitson G, Robinson P, Wilke D, Kandel RA, White L, Griffin AM (2004). Radiation response: an additional unique signature of myxoide liposarcoma. Int J Radiat Oncol Biol Phys.

[13] Magierowski K, Moseley J, Lockwood G, Parent A, Euler C, Sharpe M (2008). Retrospective study of volume changes in two pathological subtypes of sarcomas using deformation image registration. Int J Radiat Oncol Biol Phys.

[14] O’Sullivan B, Griffin AM, Dickie CI, Sharpe MB, Chung PWM, Catton CN (2013). Phase 2 study of preoperative image-guided intensity-modulated radiation therapy to reduce wound and combined modality morbidities in lower extremity soft tissue sarcoma. Cancer.

[15] Thorwarth D, Low DA (2021). Technical challenges of real-time adaptive MR-guided radiotherapy. Front Oncol.

[16] Chen X, Men K, Li Y, Yi J, Dai J (2019). A feasibility study on an automated method to generate patient-specific dose distributions for radiotherapy using deep learning. Med Phys.

[17] Bertholet J, Anastasi G, Noble D, Bel A, van Leeuven R, Roggen T (2020). Patterns of practice for adaptive and real-time radiation therapy (POP-ART RT) part II: Offline and online plan adaption for interfractional changes. Radiother Oncol.

