# Supplementary material for: Management of tumor volume changes during preoperative radiotherapy for extremity soft tissue sarcoma: a new strategy of adaptive radiotherapy
Source: Radiol Oncol. 2023 Nov 30;57(4):507–15. doi: 10.2478/raon-2023-0056 (PMC10690743; doi:10.2478/raon-2023-0056)
Supplement: Supplementary file 1 — Supplementary Material Details [file raon-2023-0056_sm.pdf]

sciendo
Radiology and Oncology | Ljubljana | Slovenia | www.radioloncol.com
RADIOLOGY AND ONCOLOGY

Management of tumor volume changes during preoperative radiotherapy for extremity soft tissue sarcoma: a new strategy of adaptive radiotherapy

Marion Geneau De Lamarliere, Amélie Lusque, Justine Attal Khalifa, Vincent Esteyrie, Christine Chevreau, Thibaud Valentin, Dimitri Gangloff, Thomas Meresse, Louis Courtot, Philippe Rochaix, Bérénice Boulet, Eliane Graulieres, Anne Ducassou

doi: 10.2478/raon-2023-0056

Appendix

To illustrate the variability in tumor behaviors during preoperative radiotherapy and its dosimetric consequences, the following graphs from each of our 17 patients show tumor dimension and volume changes, and PTV coverage repercussions during the course of RT.

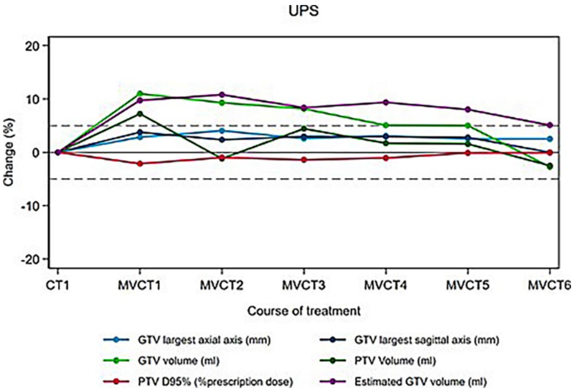

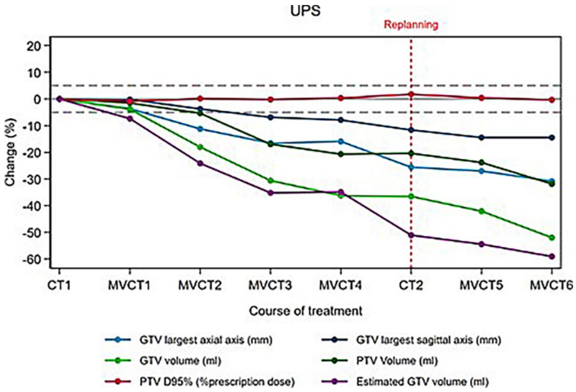

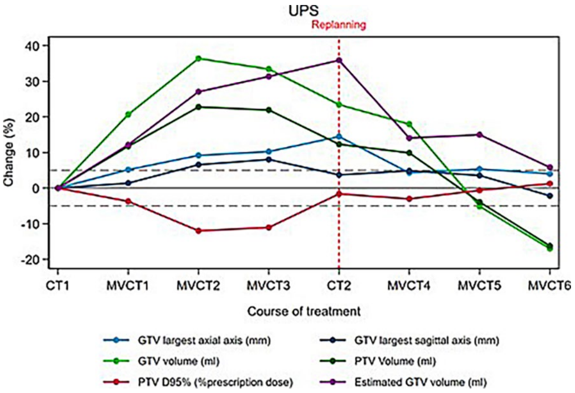

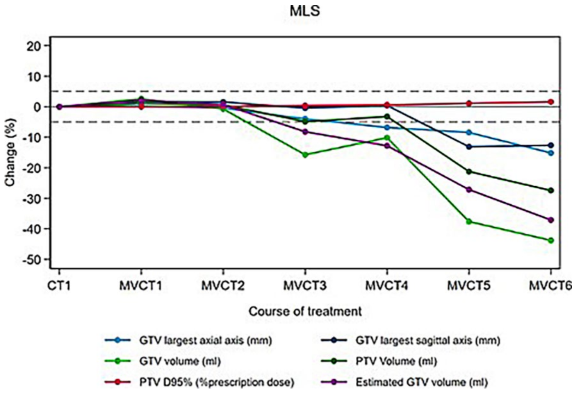

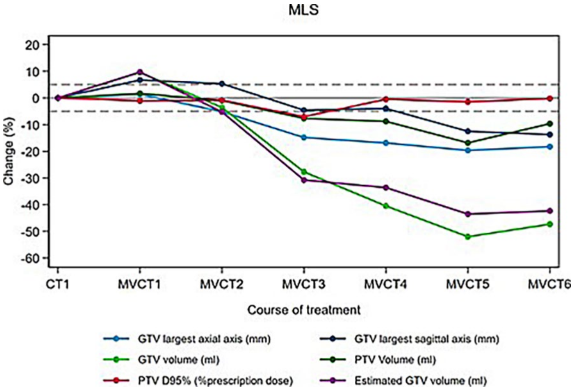

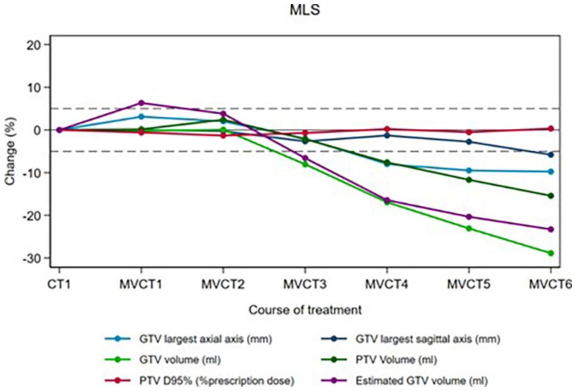

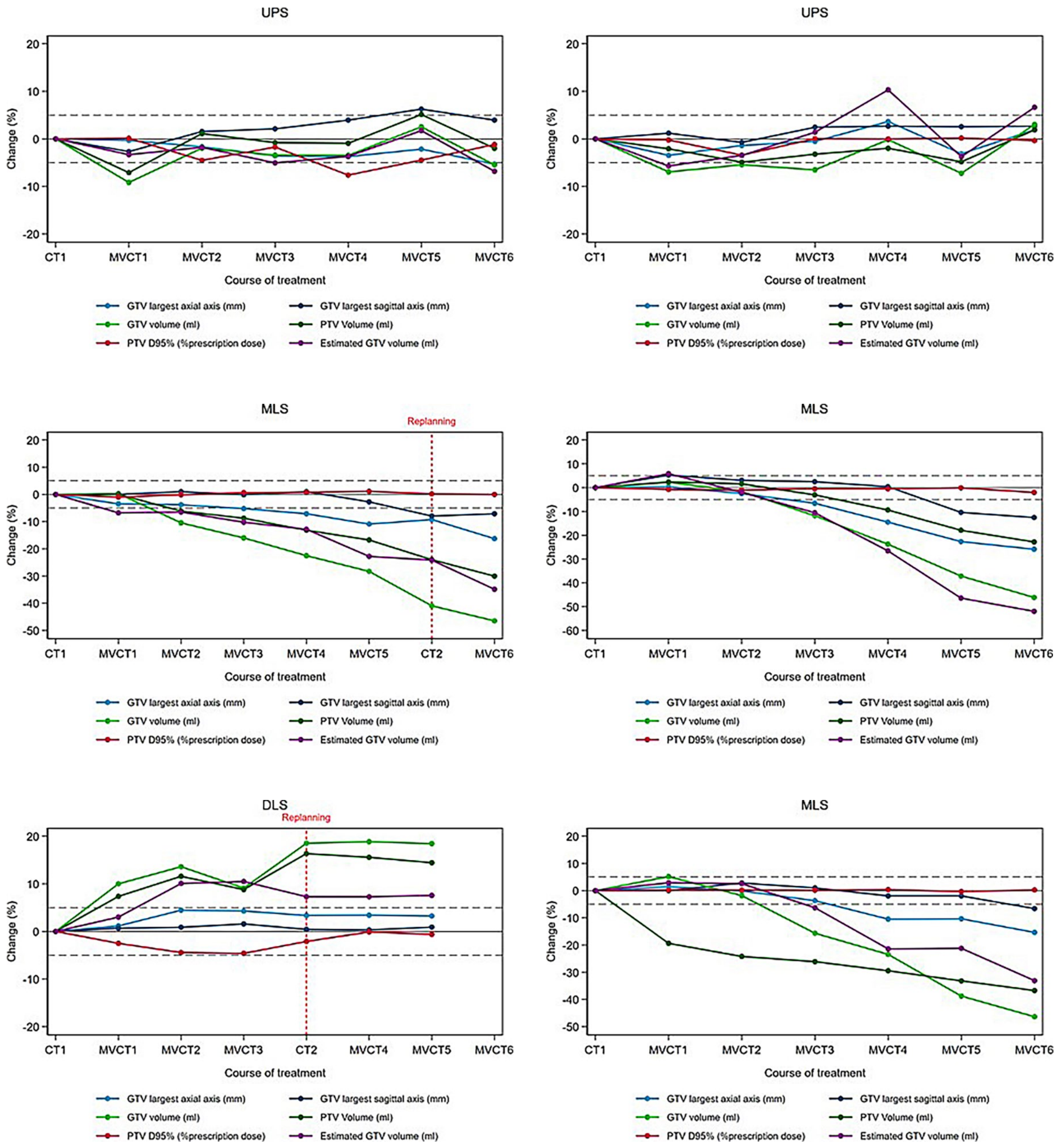

* Post plan adaptation data are not available for this patient because of a software malfunction. More MVCT than other patients have been analyzed before CT2, 42 Gy was delivered at MVCT6, he had received 70 Gy in 35 fractions at the end of treatment.

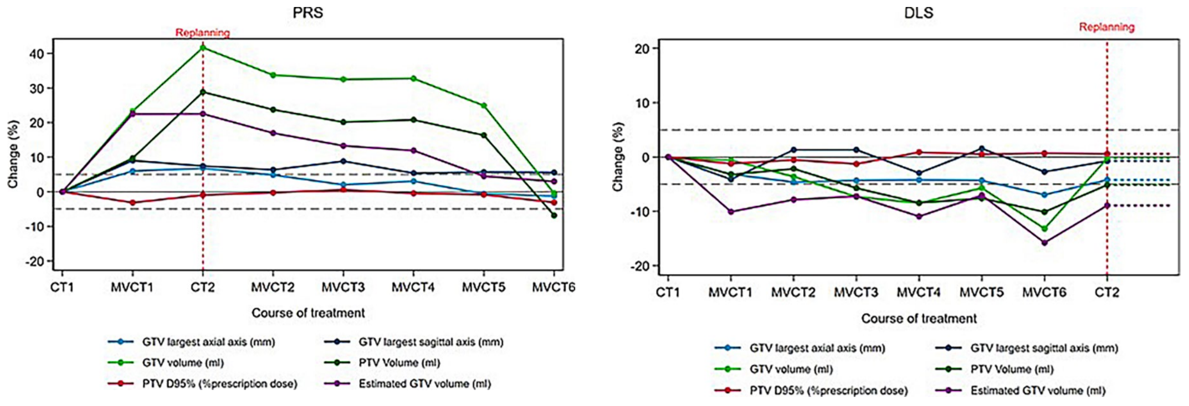

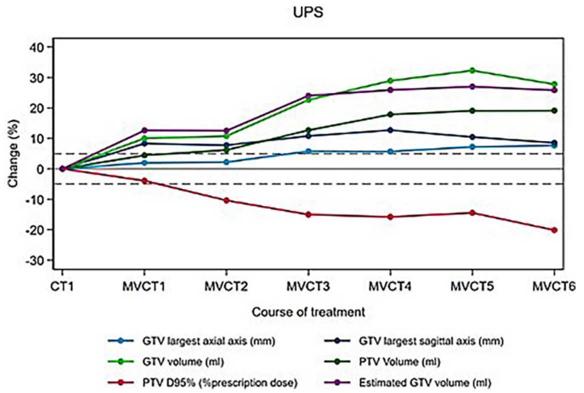

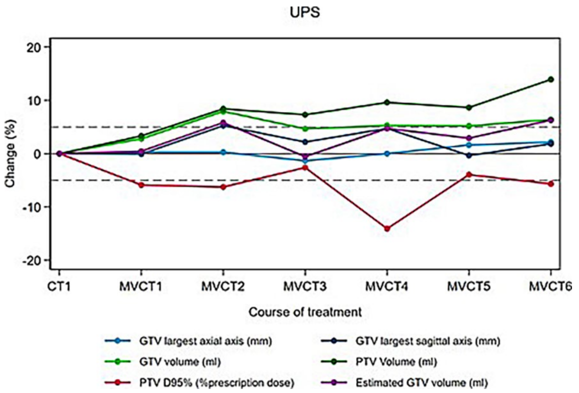

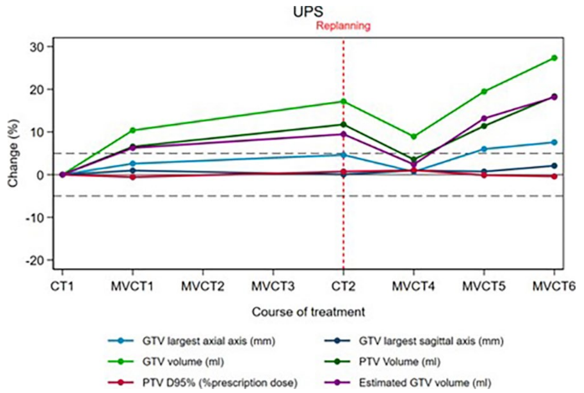

* MVCT 2 and MVCT 3 data are not available because of a software dysfunction.

DLS = dedifferentiated liposarcomas; GTV = gross tumor volume; MVCT = megavoltage computed tomography; MLS = myxoid liposarcomas; PRS = pleomorphic rhabdomyosarcoma; PTV = planned target volume; UPS = undifferentiated pleomorphic sarcomas
